# Graphite Equivalent Evaluation of Anthracite-Associated Graphite by Raman Spectroscopy

**DOI:** 10.3390/ma16237278

**Published:** 2023-11-22

**Authors:** Wubo Chu, Wen Dai, Bo Wang, Chen Ye, Weiping Xie, Bing Yin, He Li, Nan Jiang

**Affiliations:** 1 Key Laboratory of Marine Materials and Related Technologies, Zhejiang Key Laboratory of Marine Materials and Protective Technologies, Ningbo Institute of Materials Technology and Engineering, Chinese Academy of Sciences, Ningbo 315201, China; 2 Center of Materials Science and Optoelectronics Engineering, University of Chinese Academy of Sciences, Beijing 100049, China; 3 Chair of Functional Materials, Department of Materials Science & Engineering, Saarland University, 66123 Saarbrücken, Germany; 4 Dalian Customs, Dalian 116001, China

**Keywords:** anthracite-associated graphite, graphite equivalent evaluation, Raman

## Abstract

Anthracite-associated graphite is an important graphite resource with a wide range of applications besides being used as a fuel. This paper introduces a method for evaluating the graphite equivalent evaluation of anthracite-associated graphite. A series of graphite-anthracite standard samples with known graphite content were prepared, and their Raman spectra were obtained using a Raman spectrometer. By employing peak-fitting analysis to decipher the peak spectrum information of the D peak and G peak, trends in the peak position, peak intensity ratio, half-width, and peak area of the D peak and G peak in standard samples with different graphite contents were obtained. Subsequently, a standard curve and fitting equation were established using the peak area data. The goodness of fit for the equation (R^2^) was 0.9984. Then the equation was used to evaluate 100 natural anthracite-associated graphite samples with unknown graphite content, obtaining a corresponding graphite equivalent evaluation.

## 1. Introduction

The formation process of anthracite can be roughly divided into four stages: peat, lignite, bituminous stage, and anthracite [1,2,3]. Over time, anthracite coal is continuously affected by upper pressure and geothermal energy, in which the aromatic carbon polycondensation fragments are mutually bonded and synthesized into a graphite structure with a crystallite size for forming the anthracite-associated graphite [4,5,6]. As a secondary product of anthracite, the associated graphite of anthracite has new value in industrial society and scientific research and different to anthracite [7,8]. However, how to measure the graphite evaluation in anthracite-associated graphite is a difficult task.

The amorphous carbon and graphite in the anthracite-associated graphite are both made of carbon. It is challenging to measure the graphite evaluation in the anthracite-associated graphite via chemical composition analysis due to the fact that the chemical composition of the two is the same; only the crystallinity of the carbon is different [9,10]. In principle, techniques such as Raman spectroscopy, X-ray diffraction (XRD), and transmission electron microscopy (TEM) can be used for structural characterization to distinguish amorphous carbon from graphite. TEM is the most intuitive analysis tool capable of obtaining the carbon structure morphology at the micro- and nanoscale. XRD is a commonly used technique for analyzing the crystal structure of carbon materials. X-ray photoelectron spectroscopy (XPS), which analyzes the functional groups and valence states on the carbon surface, is also an auxiliary analytical technique for carbon materials [11,12,13,14,15]. Wang et al. [14] conducted a detailed structural analysis of Inner Mongolian lignite using XRD and HRTEM. The structural parameters determined by XRD indicate that the molecular structure of low-rank lignite is loosely organized, with d002 calculated as 4.56 Å and Lc as 13.59 Å. Li et al. [16] reported that in the carbon structural transformation from anthracite to natural coal-based graphite, the XRD diffraction pattern of the anthracite exhibits a broader (002) peak, while the (002) peak of semi-graphite and coal-based graphite becomes narrower. The classification of different coal-based carbon materials can be effectively achieved based on the half-width value (FWHM) of the (002) peak. Wang [14] and Li et al. [16] also reported on the application of TEM in the classification of coal-based carbon materials. HRTEM observations in lignite and anthracite samples primarily reveal an amorphous carbon structure, relatively ordered carbon structures can be observed in semi-graphite, while highly graphitized coal-derived graphite displays straight graphene layers stacked with tens of nanometers. Liu et al. [17] investigated the changes in surface carbon groups and sulfur transformation of two types of natural coal after pyrolysis under different atmospheres and temperatures using XPS. The study indicates the variation trends in C-C and C=O groups on the coal surface before and after pyrolysis. As a detection method that reflects molecular structural fingerprint information, Raman spectroscopy is of great importance in the field of structural analysis of coal-based materials due to its simplicity in sample preparation, fast testing speed, and high accuracy [18,19]. Liu et al. [20] studied the thermal degradation process of Australian bituminous coal using a Raman spectroscopy in the temperature range of 298–1473 K. The peak position, full width at half maximum (FWHM), and relative peak area in the Raman spectra reflect the changes in the carbon structure during coal thermal treatment. Rantitsch et al. [21] conducted identification studies of semi-graphite and graphite using a Raman spectroscopy. They performed peak fitting analysis on the Raman spectra of multiple natural graphite samples and successfully differentiated graphite from semi-graphite based on the FWHM width of the Raman G-band and the D1/(G + D1 + D2) area ratio information.

In this paper, standard anthracite and high-purity graphite are used to prepare various ratios of anthracite-graphite standard samples to serve as different graphite equivalents of anthracite-associated graphite. Then, the graphite equivalent in the natural anthracite-associated graphite was evaluated by the analysis and simulation of the Raman spectrum of the as-prepared standard samples.

## 2. Materials and Methods

### 2.1. Materials

High-purity graphite (purity was 99.95 wt%, particle size: ≤2 μm) was purchased from Shanghai Aladdin Reagents Ltd., Shanghai, China. The standard anthracite (fixed carbon content was 79.36 wt%, particle size: ≤200 μm) was purchased from Beijing Jiashi Yuhe Chemical Technology Research Institute, and ground with a mortar to over 50% particles (D50) less than 2 μm in this experiment to prepare samples (the scanning range of the Raman spectrum is approximately 2 μm). The anthracite-associated graphite samples were collected by the Dalian Customs Test Center.

### 2.2. Methods

Standard anthracite and high purity graphite each 15 g are divided into two groups, each group is divided into five copies according to the 0.6 g weight gradient. A standard sample with a graphite content of 20 wt% is prepared from 0.6 g high-purity graphite and 2.4 g standard anthracite and use the same method to prepare standard samples with graphite content of 40, 60, and 80 wt%. With the addition of standard graphite and standard anthracite samples, we have obtained a total of six standard samples; The final standard sample is obtained by mixing in a high-speed mixer at a speed of 2000 r/min for 5 min.

A confocal micro-Raman spectrometer (inVia Reflex, Renishaw, British) was used to obtain Raman spectra. The laser wavelength used was 532 nm, and the laser power was set to 24 mW. The scanning range was approximately 2 μm, and it took 10 s to complete a single scan. The scanning range for Raman shifts was set between 800 and 2000 cm^−1^. Each standard sample was randomly tested at least 15 times at different positions. For the associated graphite samples in anthracite coal, each sample was randomly tested at least 5 times at different positions.

Much of the literature has shown that the Raman spectra of graphite and anthracite contain multiple characteristic peaks. The G peak (1580 cm^−1^) serves as the ordered peak corresponding to the E_2g_ vibration mode, reflecting the symmetric stretching vibration of the sp^2^ hybrid carbon lattice site in the carbon material structure (ring or chain). The D1 peak (1350 cm^−1^) exists in anthracite, defective or small-sized graphite, and it is the disordered peak corresponding to the A_1g_ vibration mode, reflecting the breathing vibration of sp^2^ hybrid carbon at the ring lattice site. The D2 peak is positively correlated with the turbulence inside the graphite planar structure; the D3 peak is positively correlated with the external defects of aromatic structures like tetrahedral carbon; the D4 peak is attributed to disordered graphite lattice (A_1g_ symmetry) or ionic impurities [21,22,23,24,25,26,27,28]. These characteristic peaks can accurately reflect the characteristic structure information of graphite associated with anthracite.

This paper conducts peak fitting analysis on the Raman spectra of standard samples with different graphite contents. The inVia Reflex confocal micro-Raman spectrometer is used to calibrate the spectral baseline to mitigate the impact of varying degrees of spectral shifts caused by factors such as particle smoothness and Raman fluorescence. This calibration ensures accurate subsequent analysis [29]. Three fitting models: Lorentz, Gaussian, and Lorentz-Gaussian mixture were chosen to fit the peak spectrum with as few fitting curves as possible. After fitting the peak spectra, the spectral information of the D peak and G peak was interpreted. This analysis revealed the changes in the D peak position, peak intensity ratio, half-width, and the areas of the D and G peaks in the standard samples. Subsequently, a standard curve and fitting equation were established using the peak area data to evaluate the graphite equivalent of coal-associated graphite.

## 3. Results and Discussion

### 3.1. Data Preprocessing

**Figure 1:**
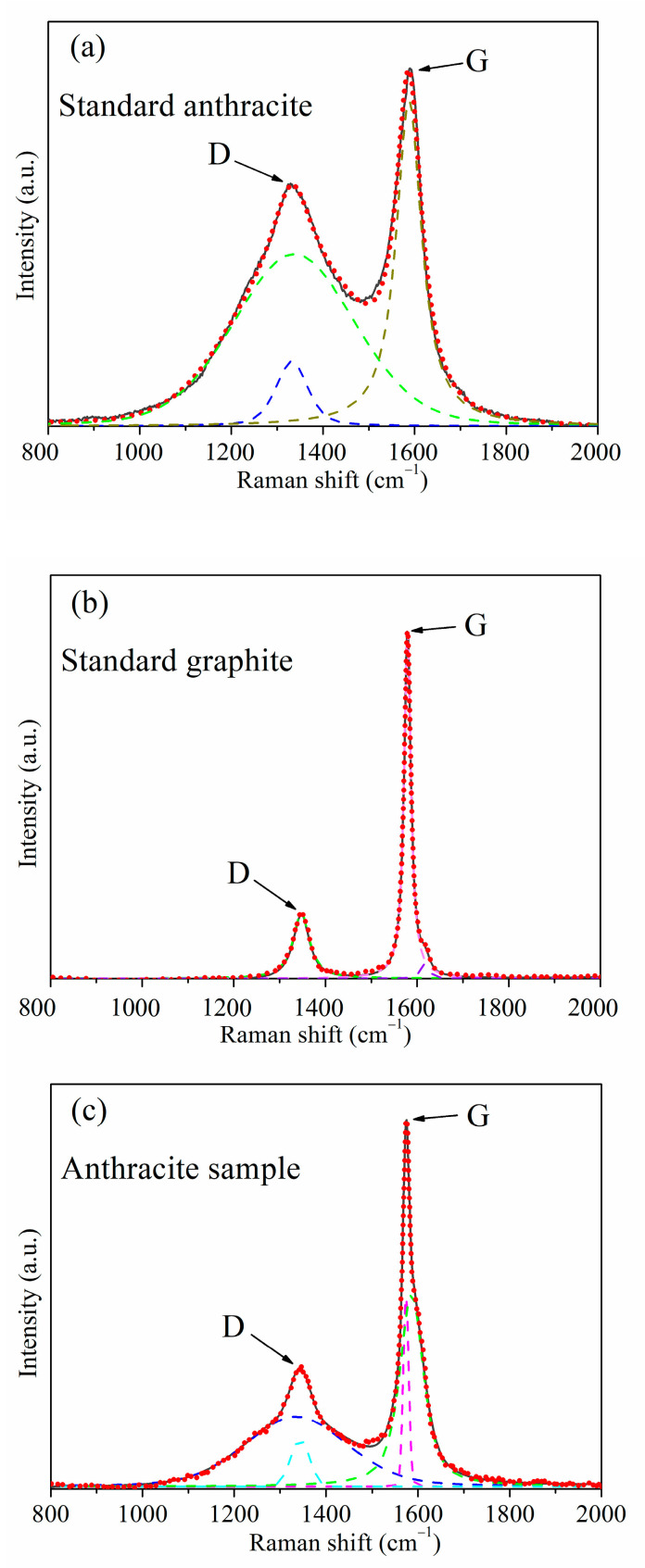
Common three peak types and fitting results graphs in the samples: (**a**) standard anthracite, (**b**) standard graphite, (**c**) anthracite sample.

Figure 1 illustrates the typical peak shapes and fitting results for different samples, The solid black line represents the original characteristic spectrum, while the red dashed line represents the fitting curve. The spectrum in Figure 1a corresponds to the characteristic curve of standard anthracite, with the D peak being higher and the G peak being relatively symmetrical. In Figure 1b, the peak spectrum exhibits a weaker D peak, and the G peak is noticeably asymmetric (with a distinct D2 peak near 1620 cm^−1^), indicating the presence of defects or smaller crystal sizes in the graphite. Figure 1c combines the peak characteristics of both anthracite and graphite, with a weaker D peak than standard anthracite but stronger than graphite. The G peak is narrower at the top and significantly asymmetric. This type of peak shape is commonly found in standard samples with graphite content ranging from 20 to 80 wt% and is also prevalent in coexisting anthracite-graphite samples. In Figure 1a, it can be seen that using a double D peak (the green and blue dashed lines) and a single G peak (the dark yellow dashed line) three-curve fitting can effectively fit the curve, with the D peak position generating a sharper small peak matching the sudden narrowing of the D peak. A single peak fitting (the green dashed line) is sufficient for the D peak position to achieve an ideal fit in Figure 1b. The asymmetric peak shape of the G peak requires a two-peak fitting (the violet and dark yellow dashed lines), so a single D peak and double G peak three-curve fitting is used. In Figure 1c, the curves combine the peak shape characteristics that require double-curve fitting from Figure 1a,b, so a four-curve fitting with double D peaks (the cyan and blue dashed lines) and double G peaks (the violet and green dashed lines) is applied. The high degree of alignment between the fitted curves and the original curves in all three figures indicates that all three fitting methods have achieved desirable fitting results.

### 3.2. Characteristic Peak Analysis of Standard Samples

**Figure 2:**
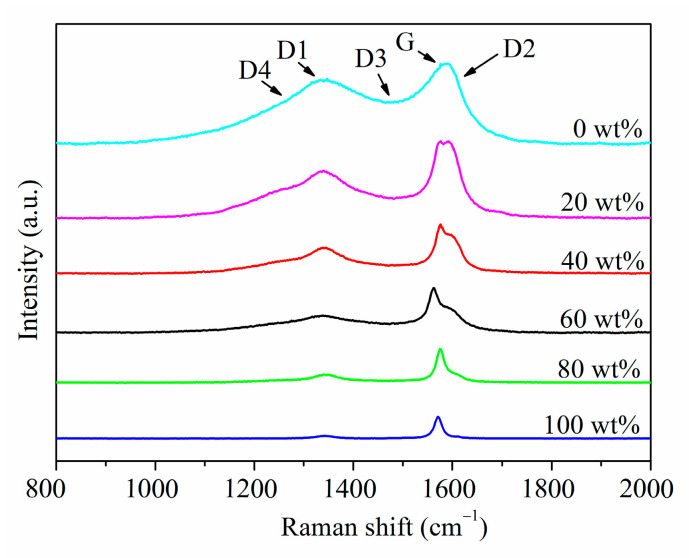
Typical Raman spectra of standard samples with different graphite contents.

This research mainly aims to establish the anthracite-graphite standard equation and analyze the graphite evaluation in anthracite-associated graphite. By preparing anthracite-graphite standard samples, acquiring at least 15 Raman spectra at different positions of each sample, extracting characteristic information after preprocessing statistical peak spectrum information and trend analysis, can then establish an A_D_/(A_D_ + A_G_) semi-quantitative standard equation between graphite content. Based on the established standard equations, the anthracite-associated graphite samples were detected to perform Raman spectroscopy, as the results shown in Figure 2, which exhibits the statistical characteristic peaks. Then qualitative and semi-quantitative methods were used to analyze the graphite content.

Figure 2 revealed that the Raman characteristic peaks of the standard samples with different graphite content increase successively from 0 to 100 wt%. When the graphite content is 0%, the D peak is very broad. As the graphite content increases, the D peak near 1350 cm^−1^ gradually becomes narrower. Contrasting the spectra at graphite contents of 20% and 40%, the D peak width continues to decrease. When the graphite content is 60%, examining the spectra at 60% and 80%, the D peak is already very weak, and the peak width continues to decrease. In high-purity graphite, we can hardly observe a distinct D peak.

**Figure 3:**
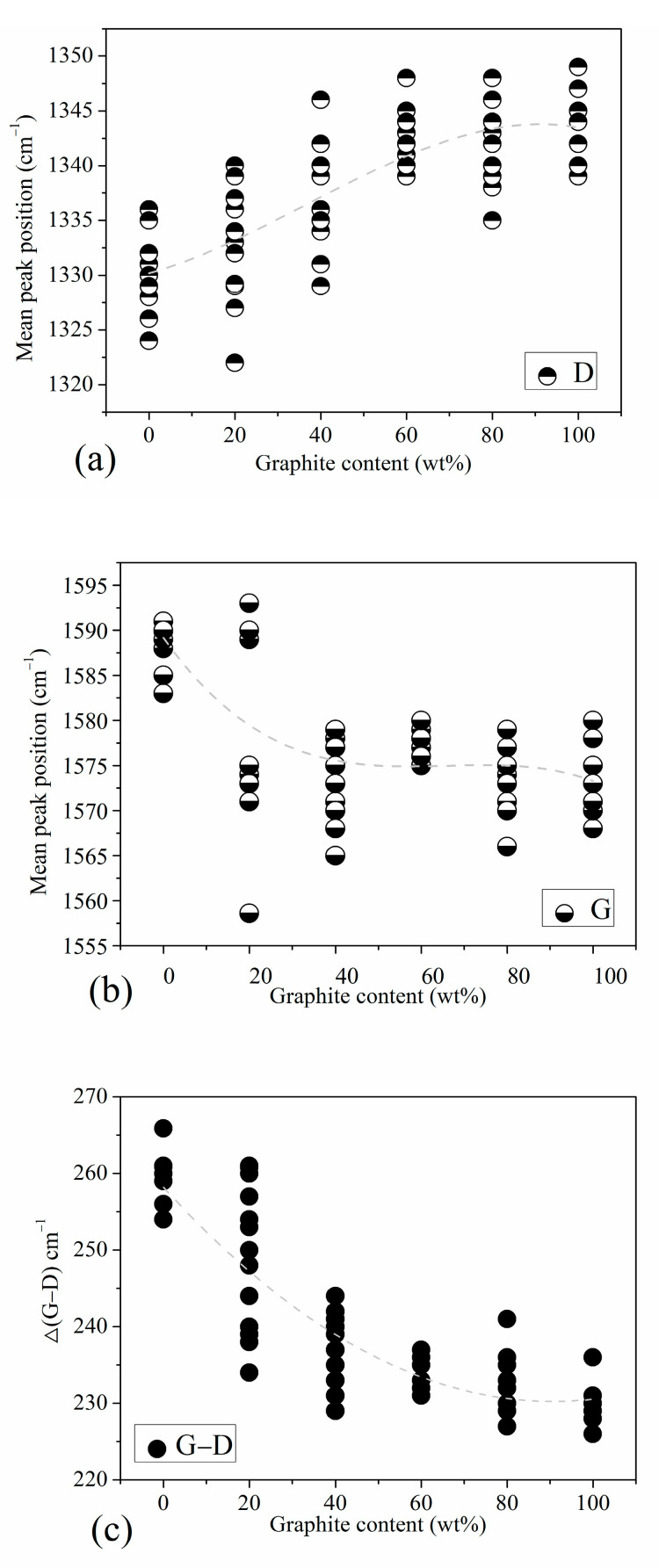
Peak position trend of standard samples with different graphite contents: (**a**) D peak position, (**b**) G peak position, (**c**) the positional difference between peak G and peak D.

We conducted a detailed analysis of the Raman spectra of the standard samples. Figure 3 shows the peak position trend graphs of standard samples with different graphite content. Combined with Figure 2, we find that the position of the D peak is actually the Raman shift value at the highest point of D1 peak. It mainly reflects the position information of the D1 peak, not greatly affected by D3 peak and D4 peak; the position of the G peak may actually reflect the displacement information of the G peak or the G peak and the D2 peak. The specific situation is related to the graphite content in the sample.

Figure 3a is the D peak position map of the standard sample, showing the corresponding relationship between the D peak position and the graphite content. The position of peak D is related to the structural disorder of coal-based carbon materials. In carbon materials with higher turbulence, such as anthracite, the D peak position is at a lower wavenumber. In high-purity graphite and other samples with a higher order of aromatic ring structure, the D peak is at a higher wavenumber. Figure 3b is the position of the G peak of the standard sample, in which, in addition to the anthracite and a small amount of 20 wt% graphite content of the standard sample, the G peak is at a higher wavenumber. The positions of most characteristic peaks of other samples are basically stable around 1573 cm^−1^. The reason why the G peak position of anthracite is at a higher wavenumber may be that its structure has a large number of fragmented and defective aromatic rings, which exhibits a very strong D2 peak, which affects the peak position of the G peak; in other words, the G at this time. The peak position is not the peak position caused by the respiratory vibration of the graphite aromatic ring structure; as the graphite content increases, the structural order of the standard samples increases successively, the order peak corresponding to the E_2g_ vibration mode of the graphite lattice begins to dominate, and the G peak position is almost no longer affected by the D2 peak. Figure 3c is a graph of the positional difference between peak G and peak D in the standard sample (Δ(G−D)); it reflects the relative positional relationship between peak G and peak D in each peak spectrum. We found that as the graphite content increases, the positions of the G peak and D peak gradually become closer. We know that this trend is mainly due to the position shift trend of the D peak with the change in graphite content.

**Figure 4:**
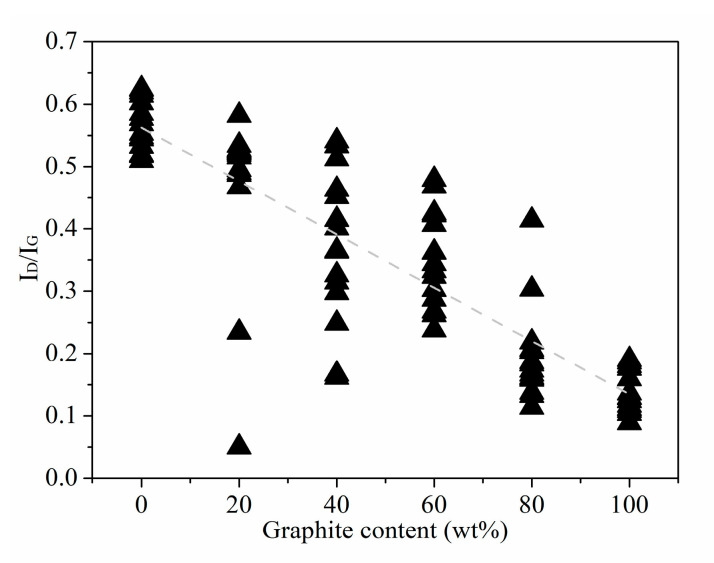
I_D_/I_G_ Trend of Standard Samples with different graphite contents.

We know that the peak intensity in the Raman of coal-based carbon materials is often characterized by the Raman intensity ratio of peak D to peak G (I_D_/I_G_). I_D_/I_G_ as an important indicator as it reflects the order of the overall structure of coal-based carbon materials in the Raman radiation area. The I_D_/I_G_ trend chart of the standard sample is shown in Figure 4. It can be seen in Figure 4 that from standard anthracite to high-purity graphite, the I_D_/I_G_ value of the standard sample basically shows a linear downward trend as the graphite content increases. This shows that with the gradual increase of graphite content according to a 20 wt% gradient, the rising rate of order in the standard sample structure is more uniform.

**Figure 5:**
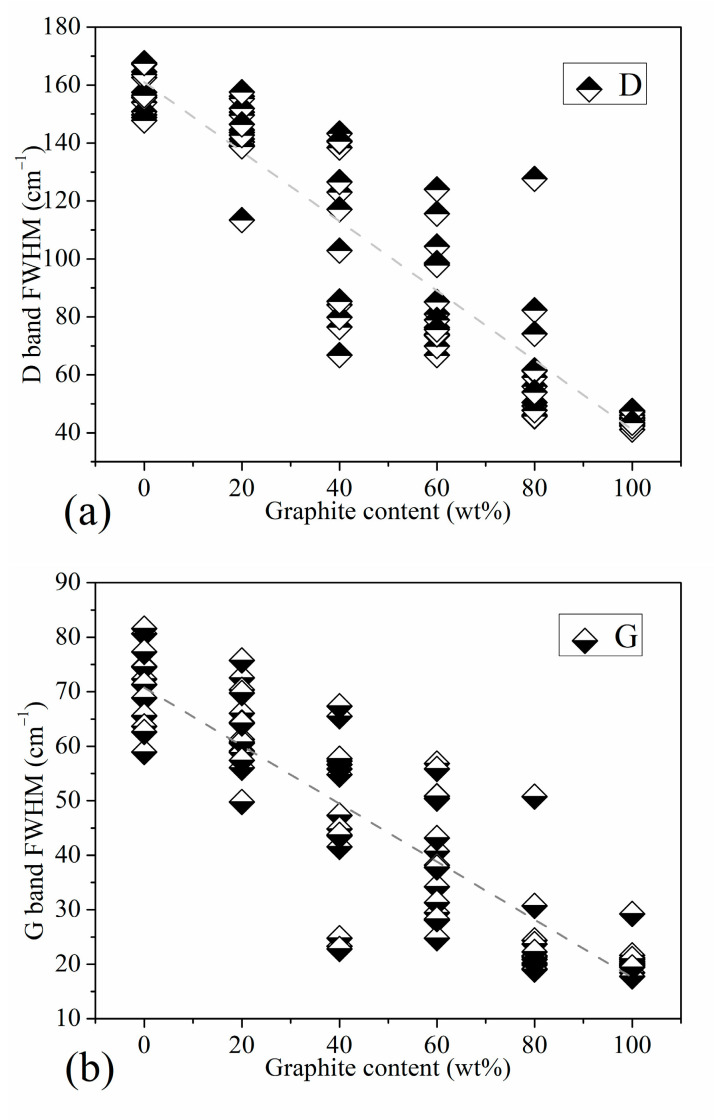
Half Peak Width Trend of Standard Samples: (**a**) the half-width of peak D, (**b**) the half-width of peak G.

Regarding the half-value width, Figure 5 shows the trend graph of the half-value width of the Raman characteristic peak of the standard sample with increasing graphite content. Since the D peak is actually a partial superposition of the three peaks of D1, D3, and D4, it is difficult to reflect on the peak position of D. Although it is reflected in the peak intensity, it is most fully reflected in the half-peak width. Figure 5a is the trend graph of the half-width of peak D. On the whole, the dispersion of the D peak half-value width of each mixed standard sample is higher than that of standard anthracite and high-purity graphite. However, it has a relatively obvious linear correspondence with the changes in the graphite content of each gradient between anthracite and high-purity graphite. This indicates that the overall peak shape of the D peak has a relatively reliable correspondence with the graphite content in the standard sample obtained via mechanical mixing. The half-width position of the ordered peak of graphite is an important indicator for judging coal rank. Figure 5b shows the trend graph of the half-value width of the G peak of our standard sample. It can be seen from the figure that when the graphite content is less than 80 wt%, the half-width of the G peak of the standard sample is basically linearly negatively correlated with the graphite content. From Figure 2, which shows the Raman characteristic peaks with graphite content of 80, 60, 40, and 20 wt%, we can see that the half-value width of the G peak is affected by the D2 peak. In addition, there is a factor that cannot be ignored that affects the half-width of the G peak. The half-width of the order peak of the graphite structure at 1580 cm^−1^ is related to the order of the crystal structure, which is also an important indicator for judging coal rank.

### 3.3. Establishment of Quantitative Relationships

**Figure 6:**
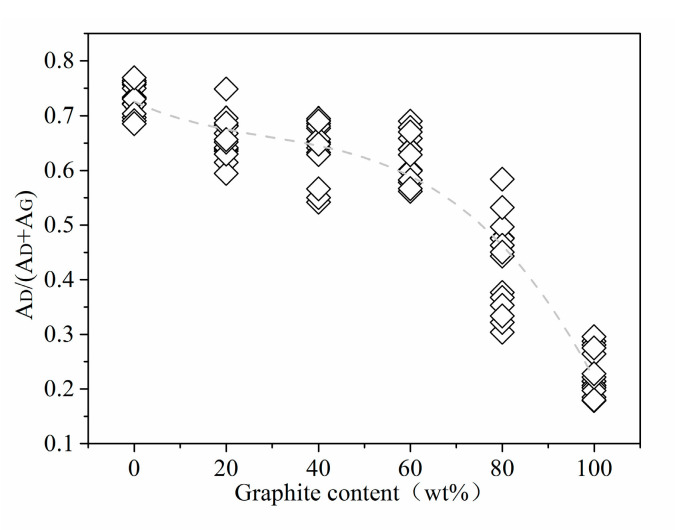
Peak area (statistics) trend of standard samples.

Peak area is also important Raman information used to analyze the structure of coal-based carbon materials. In the structure of coal-based carbon materials, the relative ratio of the sum of the areas of D1, D3, and D4 is to the sum of the areas of G and D2 (A_D_/(A_D_ + A_G_)). It reflects the proportion of defective aromatic ring carbons and ordered aromatic ring carbons in the material. Figure 6 shows the trend graph of the ratio of the D peak area of the standard sample to the total area of the D and G peaks ((A_D_/(A_D_ + A_G_)). From standard anthracite to high-purity graphite, the value of A_D_/(A_D_ + A_G_) decreases first and then quickly with the increase in graphite content. The A_D_/(A_D_ + A_G_) value of standard anthracite is the highest, and the statistical value is in the interval of 0.69–0.77. As the graphite content increases, the relative content of anthracite decreases, the specific gravity of the carbon structure with good crystal structure in the Raman scanning area increases, and the defective six-membered ring carbon structure is reduced. As a result, the value of A_D_/(A_D_ + A_G_) gradually decreases until it reaches the lowest level in high-purity graphite.

According to the data statistics of the standard sample Raman peak, the characteristic parameters, including peak height, peak position, half-peak width, and peak area have a certain correspondence with the graphite content in the standard sample. These correspondences are, ultimately, caused by different types of coal-based carbon. The material structure is caused by the difference in the signal of the scattered light under the irradiation of the Raman laser. Among the three elements of peak intensity, half-width and peak area, the peak area integrates the peak intensity and half-width information, so the peak area is used to quantitatively analyze the relationship between the Raman characteristic peaks of coal-based carbon materials and the graphite content. Therefore, this section selects the peak area information to establish the quantitative relationship between the graphite content of the standard sample and the Raman characteristic peak. The preceding analysis focuses on evaluating the trend relationship between characteristic peak information and graphite content. In order to show the corresponding relationship between the two amounts more clearly, the amounts of high-purity graphite (99.95 wt%) and standard anthracite (79.36 wt%) are counted as graphite content and non-graphite content, respectively. The conversion formula is as follows, *M* is graphite content, *N* is the aforementioned high purity graphite content:

(1) $$M=\frac{N\times 0.9995}{N\times 0.9995+(100-N)\times 0.7936}$$

We select 5 points around the median of 15 statistical values of each standard sample and calculate the average value of the 5 numerical values as the A_D_/(A_D_ + A_G_) value. Figure 3 shows the corresponding relationship between the graphite content of the standard sample and A_D_/(A_D_ + A_G_). We perform a 2-variate polynomial fit between the graphite content value and the A_D_/(A_D_ + A_G_) value. The fitting equation is as follows:

(2) $$y=-2\times 10^{-6}x^{3}+2\times 10^{-4}x^{2}-5.9\times 10^{-3}x+0.7417$$

In the formula, y is A_D_/(A_D_ + A_G_), and χ is the graphite content value; the equation goodness of fit: R^2^ = 0.9984.

For semi-quantitative analysis of graphite content in anthracite-associated graphite, we convert the corresponding relationship of Formula (2). Formula (2) is the corresponding relationship formula, where A_D_/(A_D_ + A_G_) is the y value and the graphite content is the x value. It is worth noting that the graphite content analysis process of the anthracite associated graphite is usually based on the Raman spectrum information to obtain the A_D_/(A_D_ + A_G_) value of the anthracite associated graphite to evaluate its graphite content, so we analyze the two variables in Equation (2), performing position transformation, and the result is shown in Equation (3):

(3) $$Y=-{1706X}^{3}+1899.7X^{2}-717.91X+179.05$$

Table 1 presents the average A_D_/(A_D_ + A_G_) and graphite equivalent values for 100 natural anthracite coal-associated graphite samples. The graphite equivalent values were obtained using Equation (3), with the average A_D_/(A_D_ + A_G_) as input. Among these samples, 82 had graphite equivalent values greater than 80%, indicating a high degree of graphitization. In addition, Table 1 provides the relative standard deviation (RSD) for each sample based on the original A_D_/(A_D_ + A_G_) values (three valid original A_D_/(A_D_ + A_G_) values were taken for each sample). Overall, there is a clear negative correlation between the RSD and the graphite equivalent. Among the samples with graphite equivalent values greater than 80%, 46 of them had RSD values below 1%, accounting for a certain percentage, while 2 had RSD values higher than 10%. Among the 18 samples with graphite content lower than 80%, none of them had RSD values below 1%, and 7 of them had RSD values higher than 10%. This suggests that the graphite equivalent standard equation is more suitable for the detection of graphite samples associated with high graphite content in anthracite coal. When analyzing the data in Figure 6, it is evident that the occurrence of high RSD values might be related to the differential decline rates of A_D_/(A_D_ + A_G_) in various graphite content ranges.

## 4. Conclusions

We set the graphite content of high-purity graphite to 100 wt%, and standard anthracite graphite content to 0 wt%. Based on the graphite content of high-purity graphite and standard anthracite as raw materials, the graphite content is 0, 20, 40, 60, 80, and 100 wt% standard samples. A Confocal Raman Microscope was used to collect Raman spectra of standard samples and 100 copies of anthracite-associated graphite with unknown graphite evaluation. Based on the analysis results, a polynomial fitting was performed on the corresponding relationship between the peak area and the graphite content, and based on this, the graphite content of 100 parts of anthracite-associated graphite was semi-quantitatively measured. The observation from the investigation concludes the following details:

As the graphite content increases successively, the Raman characteristic peak of the standard sample shows a good corresponding relationship with the graphite content; the D peak position obviously moves to the high wavenumber direction in the range of graphite content 0–60 wt%, G peak. The position of 0–20 wt% obviously moved to the direction of the low wavenumber. The positional difference between peak G and peak D presents a rapid and then slow downward trend; the peak intensity ratio of peak D to peak G basically decreases linearly; the half-width of peak D and peak G also basically decreases linearly.

Peak splitting fitting to obtain information on the Raman spectra of the carbon material, including the D peak areas of peaks D1, D3, and D4, the G peak area of the ordered peak of graphite, and the D2 peak. The results showed that the proportion of peak D in the sum of the areas of peak D and peak G decreased first slowly and then quickly with the increase in graphite content.

The corresponding relationship between the average value of A_D_/(A_D_ + A_G_) and the graphite content was calculated using several peak area values at the median of the peak area statistical data. The relationship between peak area and graphite content displayed by A_D_/(A_D_ + A_G_) statistical information is well expressed, and a binary cubic polynomial ideally fits the correspondence.

Based on the corresponding relationship between the A_D_/(A_D_ + A_G_) value and the graphite content in the standard sample, the graphite evaluation of 100 parts of anthracite-associated graphite was measured semi-quantitatively. The results can quantify the difference in graphite evaluation between anthracite-associated graphite. The RSD values of the test results indicate that the accuracy of this standard equation in detecting samples with graphite equivalents greater than 80% is significantly higher compared with samples with graphite equivalents below 80%. This suggests that this equation is better suited for the analysis of coal-associated graphite samples with higher graphite content.

## Figures and Tables

**Table 1:** The output graphite content value of 100 natural anthracite-associated graphite samples.

Sample Number	Average A_D_/(A_D_ + A_G_)	Graphite Content (wt%)	Relative Standard Deviation (RSD)	Sample Number	Average A_D_/(A_D_ + A_G_)	Graphite Content (wt%)	Relative Standard Deviation (RSD)
A1	0.6731	36.27	19.08%	A51	0.3574	87.24	0.33%
A2	0.6537	44.97	39.57%	A52	0.4233	86.16	0.66%
A3	0.3472	87.39	0.57%	A53	0.3948	86.74	0.37%
A4	0.4966	82.09	5.78%	A54	0.3441	87.44	0.36%
A5	0.3534	87.30	0.40%	A55	0.3238	87.85	0.45%
A6	0.3673	87.11	0.45%	A56	0.3544	87.29	0.31%
A7	0.3581	87.23	0.20%	A57	0.4893	82.74	6.38%
A8	0.3747	87.02	0.36%	A58	0.4392	85.66	19.62%
A9	0.2620	90.68	3.31%	A59	0.5773	69.49	74.57%
A10	0.4000	86.65	0.08%	A60	0.4510	85.18	35.09%
A11	0.6470	47.75	9.80%	A61	0.3427	87.47	8.79%
A12	0.5941	65.31	10.24%	A62	0.3372	87.56	0.19%
A13	0.6686	38.38	29.68%	A63	0.3534	87.30	0.31%
A14	0.6098	60.83	5.84%	A64	0.4706	84.11	0.66%
A15	0.5035	81.42	8.13%	A65	0.3654	87.14	0.24%
A16	0.6293	54.42	21.36%	A66	0.3851	86.88	0.50%
A17	0.5077	80.98	3.03%	A67	0.4902	82.67	3.80%
A18	0.5888	66.70	2.72%	A68	0.4902	82.67	3.27%
A19	0.6020	63.12	17.73%	A69	0.4686	84.24	0.79%
A20	0.4716	84.05	2.50%	A70	0.3277	87.76	0.26%
A21	0.4662	84.38	2.13%	A71	0.4405	85.61	2.09%
A22	0.4644	84.49	3.02%	A72	0.4137	86.39	0.58%
A23	0.4580	84.84	0.75%	A73	0.4176	86.30	0.72%
A24	0.5773	69.49	9.02%	A74	0.4463	85.38	0.80%
A25	0.4625	84.60	1.94%	A75	0.5536	74.37	1.52%
A26	0.3910	86.80	0.39%	A76	0.4898	82.70	3.41%
A27	0.4440	85.48	0.68%	A77	0.5705	71.00	5.79%
A28	0.4108	86.45	0.97%	A78	0.4718	84.04	3.54%
A29	0.4254	86.10	0.87%	A79	0.4565	84.92	4.27%
A30	0.4890	82.77	1.94%	A80	0.4640	84.51	1.36%
A31	0.4310	85.93	1.15%	A81	0.4039	86.58	0.96%
A32	0.5465	75.63	4.02%	A82	0.4592	84.78	2.06%
A33	0.5361	77.30	3.39%	A83	0.4044	86.58	0.50%
A34	0.3860	86.87	4.42%	A84	0.3801	86.95	0.91%
A35	0.4603	84.72	1.12%	A85	0.3750	87.01	0.14%
A36	0.4918	82.53	1.98%	A86	0.5154	80.10	2.97%
A37	0.5632	72.52	4.46%	A87	0.5284	78.43	3.47%
A38	0.2944	88.82	1.01%	A88	0.4067	86.53	0.53%
A39	0.2983	88.66	2.95%	A89	0.3379	87.55	0.86%
A40	0.3180	88.00	1.00%	A90	0.3383	87.54	1.11%
A41	0.3255	87.81	0.56%	A91	0.4812	83.39	3.91%
A42	0.3536	87.30	0.74%	A92	0.4216	86.20	0.41%
A43	0.3534	87.30	0.31%	A93	0.3663	87.13	0.99%
A44	0.5138	80.29	1.84%	A94	0.3655	87.14	0.80%
A45	0.3583	87.23	0.87%	A95	0.5465	75.62	4.97%
A46	0.4021	86.62	0.23%	A96	0.5033	81.44	1.93%
A47	0.3584	87.23	0.52%	A97	0.4569	84.89	1.48%
A48	0.4933	82.40	1.32%	A98	0.3237	87.85	0.86%
A49	0.4247	86.12	0.51%	A99	0.4063	86.54	0.39%
A50	0.3636	87.16	0.09%	A100	0.4866	82.96	2.57%

## Data Availability

Data available within the article.
